# Impact of the COVID-19 Pandemic on Maternal Well-Being during Pregnancy

**DOI:** 10.3390/jcm11082212

**Published:** 2022-04-15

**Authors:** Rosalia Pascal, Francesca Crovetto, Irene Casas, Lina Youssef, Cristina Trilla, Marta Larroya, Alex Cahuana, David Boada, Maria Foraster, Elisa Llurba, Jordi Sunyer, Fàtima Crispi, Eduard Gratacos, María Dolores Gómez-Roig

**Affiliations:** 1 BCNatal, Fetal Medicine Research Center, Hospital Sant Joan de Déu and Hospital Clínic, University of Barcelona, 08036 Barcelona, Spain; rosalia.pascal@sjd.es (R.P.); irene.casas@sjd.es (I.C.); lyoussef@clinic.cat (L.Y.); larroya@clinic.cat (M.L.); alex.cahuana@sjd.es (A.C.); dboada@clinic.cat (D.B.); fcrispi@clinic.cat (F.C.); eduard.gratacos@sjd.es (E.G.); lola.gomezroig@sjd.es (M.D.G.-R.); 2 Primary Care Interventions to Prevent Maternal and Child Chronic Diseases of Perinatal and Development Origin, RD21/0012/0001, Instituto de Salud Carlos III, 28029 Madrid, Spain; ellurba@santpau.cat; 3 Institut de Recerca Sant Joan de Déu, 08950 Barcelona, Spain; 4 Center for Biomedical Network Research on Rare Diseases, 28029 Madrid, Spain; 5 Department of Obstetrics and Gynecology, Hospital de la Santa Creu i de Sant Pau, Universitat Autònoma de Barcelona, 08041 Barcelona, Spain; ctrilla@santpau.cat; 6 ISGlobal, 08003 Barcelona, Spain; maria.foraster@isglobal.org (M.F.); jordi.sunyer@isglobal.org (J.S.); 7 Institut de Recerca August Pi Sunyer, 08036 Barcelona, Spain

**Keywords:** COVID-19, SARS-CoV-2, pandemic, well-being, pregnancy, psychiatric disorders, anxiety, depression

## Abstract

The outbreak of a pandemic has negative psychological effects. We aimed to determine the impact of the SARS-CoV-2 pandemic during pregnancy and identify the risk factors for maternal well-being. A multicenter, prospective, population-based study was carried out that included women (*n* = 1320) who were pregnant during the SARS-CoV-2 pandemic in Barcelona (Spain) compared against a pre-pandemic cohort (*n* = 345). Maternal well-being was assessed using the validated World Health Organization Well-Being Index Questionnaire (WHO-5 Index). Pregnant women attended during the COVID-19 pandemic showed worst WHO-5 well-being scores (median (IQR) of 56 (36–72) for the pandemic cohort vs. 64 (52–76) for the pre-pandemic cohort *p* < 0.001), with 42.8% of women presenting a poor well-being score vs. 28% for the pre-pandemic cohort (*p* < 0.001). Presence of a previous psychiatric disorder (OR 7.1; 95% CI 2.6–19, *p* < 0.001), being in the third trimester of pregnancy (OR 1.7; 95% CI 1.5–2, *p* < 0.001), or requiring hospital admission for COVID-19 (OR 4.7; 95% CI 1.4–16.7, *p* = 0.014), significantly contributed to low maternal well-being during the COVID-19 pandemic (multivariate analysis). Being infected by SARS-CoV-2 was not associated with a lower well-being score. We conclude that, during the COVID-19 pandemic, there were higher rates of poor maternal well-being; the infection of SARS-CoV-2 itself did not worsen maternal well-being, but other factors as psychiatric disorders, being in the third trimester of pregnancy or hospital admission for COVID-19 disease did.

## 1. Introduction

Severe acute respiratory coronavirus 2 (SARS-CoV-2) is a global challenge for healthcare sectors and individuals. Since the outbreak, many countries have adopted strict measures, such as lockdowns, aimed at mitigating the spread of the disease [1]. Previous evidence has revealed the negative psychological impact, in terms of anxiety, depression, and post-traumatic stress symptoms [2,3] associated to the outbreak of a pandemic and its consequences on the general population, particularly on people who have quarantined [3,4].

The coronavirus 19 disease (COVID-19) has been widely studied in pregnant women. Mostly, pregnant women with SARS-CoV-2 infection remain asymptomatic and the overall rate of complications has been found to be similar to that of non-infected women [5], except close to delivery in the third trimester, where the rate of complications increases [5,6,7]. However, this population might still be vulnerable to medical and social risks [8]. Changes in preventive health-care seeking behavior due to lockdown and healthcare policies (prenatal care and pregnancy follow-up) may increase pregnancy-related stress disorders, have a negative effect on well-being, increase the risk of post-partum depression, and exacerbate other mental health problems [4]. During the initial spread of COVID-19 in 2020, pregnant women had less prenatal visits, relatives were not allowed to attend prenatal and postnatal visits, there was uncertainty regarding fetal transmission, and strict health measures led to social isolation [9].

Many studies have assessed the negative impact of the pandemic on maternal psychological status during pregnancy [10,11,12], but few studies have compared this impact to a previous pre-pandemic cohort [13] based on laboratory confirmation of SARS-CoV-2 infection [14,15,16,17]. Most current published studies on maternal psychological impact of the COVID-19 pandemic focus on depressive disorders, mental stress, or anxiety, leaving maternal well-being aside. Assessing well-being may provide a better and more general picture of the impact the pandemic has on the physical and psychological status during pregnancy.

It remains unclear whether the impact on maternal well-being is related to the COVID-19 infection itself, its severity, symptomatology, or if it is secondary to pandemic lockdown and social restrictions. The aim of this study was to examine the impact of the COVID-19 pandemic and lockdown on maternal well-being during pregnancy and identify its risk factors.

## 2. Materials and Methods

### 2.1. Study Design and Participants

A multicenter, prospective, population-based study was carried out between March 2020 and May 2020 in Barcelona, Spain [5,18]. SARS-CoV-2 infection was confirmed in all participants by the presence of antibodies and/or real-time polymerase chain reaction (RT-PCR), as described elsewhere [5]. Inclusion criteria: pregnant women who attended the participating university hospitals (Hospital Clínic, Hospital Sant Joan de Déu, and Hospital de Sant Pau) for first/second trimester screening for Down’s syndrome (10–16 weeks of gestation) or admitted to the hospital for obstetric causes or delivery and were able to undergo a well-being assessment. Pregnant women referred for a SARS-CoV-2 diagnosis outside the catchment area of the participating centers were excluded from the study. The study was approved by the Ethics Committee of each of the participating hospitals (HCB: HCB-2020-0434, HSJD: PIC-56-20, HSP: IIBSP-COV-2020-38). All participants signed their informed consent before being included in the study.

The pandemic cohort was compared to a previous cohort of pregnant women recruited between February 2017 and October 2019 before the COVID-19 pandemic [19] (Table A1).

### 2.2. Aims of the Study

The primary purpose of the study was to evaluate maternal well-being, assessed with the World Health Organization’s Well-Being Index (WHO-5) [20]. The WHO-5 consists of a five-item scale that measures quality of life and psychological well-being based on patients’ feelings within the last 15 days. The raw score ranges from 0 to 25, 0 representing the worst possible and 25 the best possible quality of life. Women were classified according to their well-being status as having a poor (≤52) or a favorable (>52) WHO-5 score [21]. The questionnaire was self-administered at recruitment. Comparisons of well-being scores between pandemic and pre-pandemic cohorts were carried out.

The second aim of this study was to assess maternal and pregnancy variables that may act as potential risk factors for a poorer well-being status, as well as data related to SARS-CoV-2 infection, quarantine, and lockdown.

### 2.3. Data Collection

Baseline and socioeconomic characteristics (working status, housing characteristics, and availability of green areas during lockdown) were obtained from a structured questionnaire, and medical and obstetric histories from the medical records at recruitment.

COVID-19 symptoms were recorded at hospital admission using a structured questionnaire that included questions on risk factors and COVID-19 suggestive symptoms noticed between mid-February 2020 and the time of SARS-CoV-2 testing. Women who tested positive, completed the same questionnaire again 4–5 weeks later. Symptomatic SARS-CoV-2 infected women were defined as having at least one of the following symptoms: fever, dry cough, anosmia or ageusia, dyspnea, myalgia, diarrhea, sore throat, skin rash, or discoloration of fingers and/or toes. More details can be found in Appendixe A and Appendixe B.

Pregnancy, delivery, and neonatal data were obtained from electronic medical files at delivery and during the postpartum period.

### 2.4. Statistical Analysis

For the primary outcome, the analyses were based on WHO-5 scorings. Secondary analyses were assessed by comparing the cohort of women who were pregnant during the SARS-CoV-2 pandemic against the pre-pandemic group. Quantitative variables were assessed for normality using Shapiro–Wilk’s test: normally distributed variables were compared using the t-test and expressed as mean and standard deviation (SD). Non-normally distributed variables were compared using the U-Mann–Whitney test and expressed as median and interquartile range (IQR). Qualitative variables were compared using χ^2^ or Fisher’s exact tests. Logistic regression analyses were performed to assess the association between maternal well-being and potential risk factors adjusted by gestational age at recruitment. A *p*-value < 0.05 was considered as statistically significant. The analyses were performed on SPSS v26 (New York, NY, USA).

## 3. Results

### 3.1. Characteristics of the Study Population

During the pandemic, 1320 women were recruited; 444 (33.6%) were in the first trimester (median (IQR) gestational age 10.7 weeks (9.9–12.1)) and 876 (66.4%) in the third trimester (median (IQR) gestational age 39.7 weeks (38.6–40.6)) of pregnancy. Table 1 summarizes the baseline characteristics of the population and Table 2 shows pregnancy and neonatal outcomes. Most women (*n* = 851, 64.5%) had a vaginal delivery; 202 (15.3%) were positive for SARS-CoV-2 at recruitment, determined by either presence of antibodies (*n* = 200) and/or positive RT-PCR (*n* = 26) (Table A3). Table A1 summarizes the characteristics of the pre-pandemic cohort.

### 3.2. Maternal Well-Being

**Figure 1:**
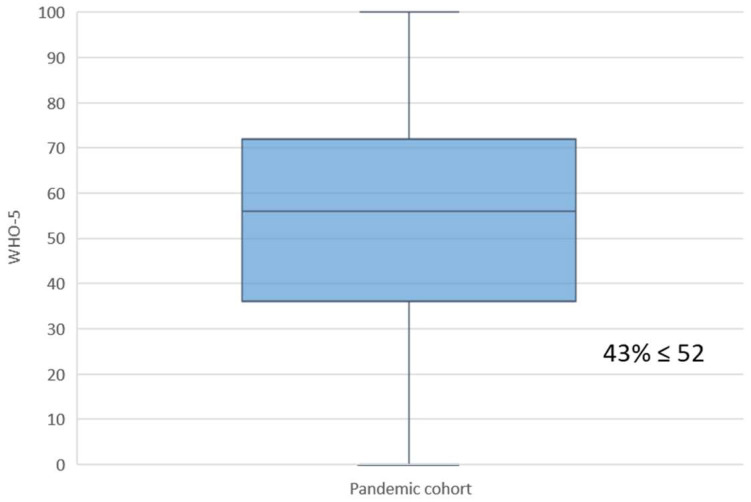
Maternal WHO-5 well-being outcomes for the pandemic cohort.

The median (IQR) WHO-5 score in the overall pandemic cohort was 56 (36–72); the score in 565 women (42.8%) was ≤52, suggestive of poor well-being, whereas in 755 participants (57.2%) it was >52, indicating favorable well-being (Figure 1).

**Figure A1:**
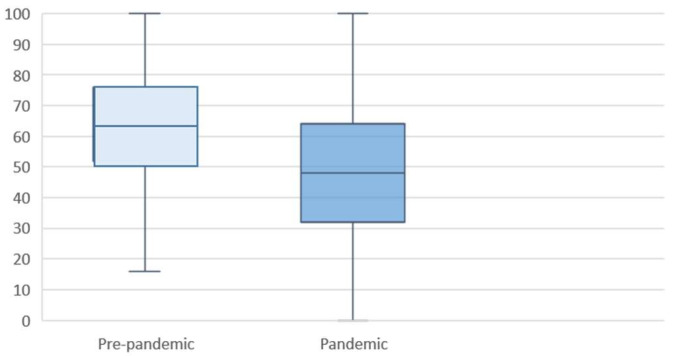
WHO-5 well-being level in pre-pandemic (*n* = 345) and pandemic (*n* = 1320) cohorts.

WHO-5 results for pregnant women during the COVID-19 pandemic (median (IQR) 56 (36–72)) were worse than for the pre-pandemic cohort (*n* = 345), (median (IQR) 64 (52–76)) (*p* < 0.001). In the pandemic cohort, 42.8% of women had a poor well-being score vs. 28% for the pre-pandemic cohort (*p* < 0.001) (Figure A1). Results were adjusted by ethnicity and psychiatric disorders (Table A1).

**Figure 2:**
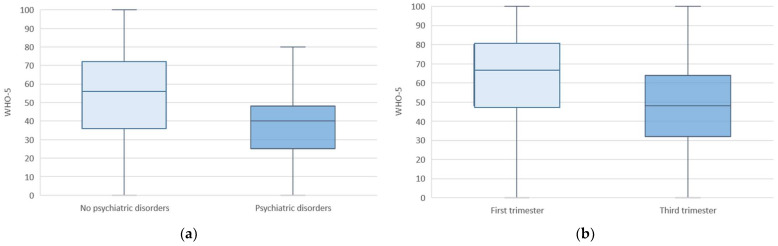
Maternal WHO-5 well-being outcomes for the pandemic cohort based on the presence of psychiatric disorders (**a**) and trimester of pregnancy (**b**).

Table 3 shows the characteristics of the COVID-19 cohort, classified according to maternal WHO-5 well-being. No significant statistical differences were found for maternal age, ethnicity, socioeconomic status, BMI, parity, or assisted reproductive technologies. However, the existence of previous maternal psychiatric disorders was a significant contributor to low maternal well-being (4.1% vs. 0.6% in case of a favorable well-being, *p* < 0.001) (Figure 2a).

Regarding pregnancy and neonatal outcomes, being in the third trimester of pregnancy was significantly associated to worse maternal well-being (median (IQR) score 48 (I32–64) (*p* < 0.001) (Figure 2b). This association was not seen for preeclampsia, prematurity, cesarean section, or fetal distress among others (Table 4).

**Figure 3:**
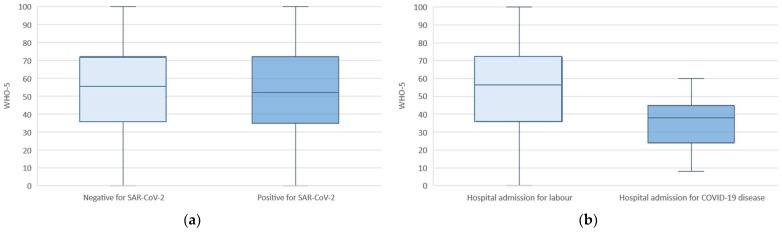
WHO-5 well-being outcomes for the pandemic cohort based on SARS-CoV-2 infectious status: (**a**) positive vs. negative women; (**b**) women hospitalized for moderate-severe COVID-19.

Regarding SARS-CoV-2 infection, the infection itself did not have an effect on the level of maternal well-being (*p* = 0.812) (Figure 3a). However, presence of severe symptoms (fever, cough, or dyspnea) and hospital admission for COVID-19 were associated with a lower well-being score (Table 5 and Figure 3b). No SARS-CoV-2 infection cases were reported in newborns.

Multivariate analyses revealed significant contribution to low maternal well-being with the presence of psychiatric disorders (OR 7.1; 95% CI 2.6–19, *p* < 0.001), being in the third trimester of pregnancy (OR 1.7; 95% CI 1.5–2, *p* < 0.001), or hospital admission for COVID-19 (OR 4.7; 95% CI 1.4–16.7, *p* = 0.014) (Table 6). No association was found between SARS-CoV-2 infection itself and a reduced well-being score.

### 3.3. Lockdown Characteristics

Four hundred and eighty participants of the pandemic cohort answered a structured questionnaire on lockdown characteristics (Table A2). Most pregnant women remained isolated in their usual residence (*n* = 448; 93.3%) without older people at home (*n* = 434; 90.4%) and the majority (*n* = 439; 91.5%) were not concerned with the general impact of the pandemic, although 332 (69.2%) communicated they were worried about their pregnancy and their fetus. No significant contributors to maternal well-being status were identified (Table A2).

## 4. Discussion

### 4.1. Main Findings

The well-being score in almost half (43%) of our study population is low. This has been related to symptoms of depression [21]. Thus, maternal well-being status during the COVID-19 pandemic is affected. This is more evident when we compare pandemic versus pre-pandemic cohorts, where 28% of the latter cohort had poor well-being scores. Additionally, there are risk factors that contribute to a worse well-being during pregnancy, such as previous psychiatric disease, being in the third trimester of pregnancy, and hospital admission for COVID-19 disease. The infection of SARS-CoV-2 itself did not increase the risk of a lower well-being condition, but the severity of COVID-19 disease requiring hospitalization did.

Well-being is broadly defined as ‘the quality and state of a person’s life’ [22] and consists of two components: feeling healthy and relatively robust and being able to carry out ones job and other tasks satisfactorily [23]. Fear related to childbirth is multidimensional and, under normal circumstances, only around 20% of pregnant women experience excessive concern regarding future events in pregnancy [23]. Feelings of well-being are key to the overall health of an individual but can be affected by physical and emotional trauma.

Several studies have reported a compromised maternal mental status during the COVID-19 pandemic [3,12,16,24]. Higher depressive rates in comparison to pre-pandemic subjects [13] and prevalence of depressive and anxiety symptoms ranging around 15–19% and 11–31%, respectively, [12,16] have been found. However, most of these works are based on maternal depression and anxiety scales and a limited number use maternal well-being as an assessment of maternal physical, mental, and social health [23].

Few studies have compared pandemic cohort data to a previous pre-pandemic cohort, suggesting worse maternal anxiety and depression levels in patients assessed during the COVID-19 pandemic. Wu et al. reported higher depression symptoms in patients during the pandemic in comparison to a pre-pandemic cohort and found a positive association with the number of newly COVID-19 confirmed cases, suspected cases, and deaths [13]. Similarly, in a study by Berthelot et al., the authors found that COVID-19 pandemic-affected women were more likely to present depressive and anxiety symptoms, especially those with a previous psychiatric diagnosis or low income [25]. Zanardo et al. reported higher scores for anhedonia and depression in comparison to 100 previous patients [26]. Interestingly, Dong et al. found that anxiety levels of pregnant women were the same as before the pandemic, while the level of depression was significantly higher. The authors reported no differences in terms of gestational age or testing positive for Sars-CoV-2 infection [17]. Perzow et al. compared 135 patients pre- and post-pandemic and determined higher levels of anxiety and depression during the pandemic [27]. To the best of our knowledge, ours is the first study that assesses maternal well-being before and after the pandemic.

Our results suggest that the existence of a previous psychiatric maternal condition is as a risk factor for worse maternal well-being. Similarly, some studies have reported that a previous psychiatric disorder diagnosed in pregnant women is as a risk factor for depression symptoms during the COVID-19 pandemic [25,28,29]. The stage of pregnancy had a unique association with anxiety and the level of well-being. Zeng et al. reported that the third trimester of pregnancy at the time of the COVID-19 pandemic seemed to be associated with a worse maternal well-being, with even worse results in comparison to the post-partum period [12]. On the contrary, Saccone et al. found worse results in anxiety and psychological impact in pregnant women in the first trimester [24]. Other authors found no differences according to gestational age [11,17,30].

COVID-19 symptoms and infection have been described as anxiety factors [31] and predictors for post-traumatic stress disorder [32]. However, these studies did not consider the differences between confirmed SARS-CoV-2 infected and healthy patients. SARS-CoV-2 infection may increase the level of anxiety and worsen mental condition; our data do not confirm this hypothesis as found in other studies with smaller sample sizes [15,17]. We report worse maternal well-being in SARS-CoV-2 infected mothers with severe symptoms or requiring hospital admission due to COVID-19 disease for respiratory and or medical support according to our center protocols at the time of the study.

### 4.2. Clinical Relevance

Our results suggest the potential utility of maternal well-being screening during the COVID-19 pandemic, especially in patients with a previous diagnosis of mental illness and in their third trimester of pregnancy, close to delivery. There is no negative effect of SARS-CoV-2 maternal infection on their well-being. However, well-being is affected in pregnant women who require hospital admission for moderate to severe COVID-19 disease, who might benefit from a psychological support during their hospital stay.

### 4.3. Strengths and Limitations

Some of the strengths of this study include a very well characterized population of pregnant women, laboratory confirmation of SARS-CoV-2 infection in all women in different pregnancy stages and during the first wave of COVID-19 pandemic, where strict restriction measures were applied. The short and simple WHO-5 questionnaire can screen depressive symptoms and evaluate subjective well-being in pregnant populations, which can be helpful in daily clinical practice, especially when healthcare pressure is high. There are several limitations to this study. The WHO-5 questionnaire was self-administration with no psychiatric screening thereafter, there were no postpartum depression or anxiety symptoms follow-ups, and baseline characteristics of the pre-pandemic and pandemic cohorts were not identical. To overcome these limitations, we applied careful statistical adjustments. Moreover, our study did not include a follow-up of postpartum depression or anxiety symptoms that could be considered in future studies.

## 5. Conclusions

In conclusion, the COVID-19 pandemic is a challenge for pregnant women in terms of well-being, especially in their third trimester of pregnancy. Previous psychiatric disorders are associated to higher risk of poor well-being. The well-being of pregnant women testing positive for SARS-CoV-2 infection is not affected, except when presenting severe infection-related symptomatology or requiring hospitalization due to COVID-19 disease, in which cases poorer well-being was reported.

## Figures and Tables

**Table 1:** Pandemic cohort baseline characteristics.

Characteristics	Total Cohort (*n* = 1320)
Age (years)	33.3 (29.1–37)
Ethnicity	
White	858 (65%)
Latin American	297 (22.5%)
Black	23 (1.7%)
Asian	81 (6.1%)
Others	61 (4.6%)
Education level	
Not educated	31 (2.3%)
Primary	86 (6.5%)
Secondary	361 (27.3%)
Vocational	191 (14.5%)
University	651 (49.3%)
Working status	
Employed	930 (70.5%)
Unemployed	262 (19.8)
Housewife	113 (8.6%)
Student	15 (1.1%)
Low socio-economic status	417 (31.6%)
Tobacco use during pregnancy	127 (9.6%)
Pre-pregnancy BMI (kg/h^2^)	24.1 (4.7)
Medical history	
Obesity (BMI > 30)	157 (11.9%)
Psychiatric disorders *	28 (2.1%)
Cardiac diseases	45 (3.4%)
Respiratory disorders	65 (4.9%)
Diabetes Mellitus	18 (1.4%)
Thyroid diseases	91 (6.9%)
Obstetric history	
Nulliparous	724 (54.9%)
Assisted reproductive technologies	98 (7.4%)

Data expressed as *n* (%), median (IQR), or mean (SD). BMI: Body Mass Index. * Psychiatric disorders requiring therapy during pregnancy.

**Table 2:** Pandemic cohort pregnancy and neonatal outcomes.

Characteristics	Total Cohort (*n* = 1320)
Preeclampsia	57 (4.3%)
Threatened/spontaneous preterm delivery	55 (4.2%)
Preterm premature rupture of the membranes	40 (3%)
Stillbirth	7 (0.5%)
Induction of labor	509 (38.6%)
Gestational age at recruitment	
In first trimester	10.7 (9.9–12.1)
In third trimester	39.7 (38.6–40.6)
Gestational age at delivery	39.2 (2.2)
Prematurity (<37 weeks)	84 (6.4%)
Mode of delivery	
Vaginal delivery	851 (64.5%)
Operative vaginal delivery	123 (9.3%)
Cesarean section	346 (26.2%)
Fetal distress	123 (9.3%)
Female gender	616 (46.7%)
Birth weight (grams)	3280 (2985–3580)
Birth weight percentile	48 (24–74)
Small for gestational age (<10th centile)	154 (11.7%)
Severe small for gestational age (<3rd centile)	52 (3.9%)
Large for gestational age (>90th centile)	157 (11.9%)
5-min Apgar 5 score	9.9 (0.7)
Neonatal complications	52 (3.9%)

Data expressed as *n* (%), median (IQR), or mean (SD).

**Table 3:** Pandemic cohort baseline characteristics based on maternal well-being (WHO-5).

Characteristics	WHO-5 ≤ 52 (*n* = 565)	WHO-5 > 52 (*n* = 755)	*p*-Value
Age (years)	32.8 (28.8–37)	33.6 (29.6–37.2)	0.050
Ethnicity			
White	367 (65%)	491 (65%)	0.977
Latin American	135 (23.9%)	162 (21.5%)	0.294
Black	6 (1.1%)	17 (2.3%)	0.102
Asian	37 (6.5%)	44 (5.8%)	0.589
Others	20 (3.5%)	41 (5.4%)	0.105
Education level			
Not educated	13 (2.3%)	18 (2.4%)	0.921
Primary	35 (6.2%)	51 (6.8%)	0.683
Secondary	168 (29.7%)	192 (25.6%)	0.092
Vocational	76 (13.5%)	115 (15.2%)	0.363
University	273 (48.3%)	378 (50.1%)	0.530
Working status			
Employed	396 (70.1%)	534 (70.7%)	0.801
Unemployed	107 (19%)	154 (20.4%)	0.520
Housewife	54 (9.6%)	59 (7.8%)	0.259
Student	7 (1.2%)	8 (1.1%)	0.761
Low socio-economic status	182 (32.2%)	235 (31.1%)	0.674
Tobacco use during pregnancy	53 (9.4%)	74 (9.8%)	0.798
BMI (kg/h^2^)	24 (4.6)	24.2 (4.8)	0.340
Medical history			
Obesity (BMI > 30)	67 (11.9%)	90 (11.9%)	0.972
Psychiatric disorders *	23 (4.1%)	5 (0.7%)	<0.001
Cardiac diseases	13 (2.3%)	32 (4.2%)	0.055
Respiratory disorders	29 (5.1%)	36 (4.8%)	0.762
Diabetes Mellitus	6 (1.1%)	12 (1.6%)	0.414
Thyroid diseases	30 (5.3%)	61 (8.1%)	0.049
Obstetric history			
Nulliparous	314 (55.6%)	411 (54.4%)	0.681
Assisted reproductive technologies	36 (6.4%)	62 (8.2%)	0.207

Data expressed as *n* (%), median (IQR), or mean (SD). BMI: Body Mass Index. * Psychiatric disorders requiring therapy during pregnancy.

**Table 4:** Pregnancy and neonatal outcomes for the pandemic cohort based on WHO-5 well-being.

Characteristics	WHO-5 ≤ 52 (*n* = 565)	WHO-5 > 52 (*n* = 755)	*p*-Value
Trimester			<0.001
First trimester	117 (20.7%)	327 (43.3%)	
Third trimester	448 (79.3%)	428 (56.7%)	
Preeclampsia	28 (5%)	29 (3.8%)	0.324
Threatened/spontaneous preterm labor	29 (5.2%)	25 (3.6%)	0.147
Preterm premature rupture of the membranes	15 (2.7%)	25 (3.3%)	0.491
Stillbirth	3 (0.5%)	4 (0.5%)	0.998
Induction of labor	226 (40%)	283 (37.5%)	0.353
Gestational age at delivery	39.1 (2.3)	39.3 (2.1)	0.316
Prematurity (<37 weeks)	40 (7.1%)	44 (5.8%)	0.357
Mode of delivery			
Vaginal delivery	361 (63.9%)	490 (64.9%)	0.705
Operative vaginal delivery	56 (9.9%)	67 (8.9%)	0.551
Cesarean section	148 (26.2%)	198 (26.2%)	0.990
Fetal distress	61 (10.8%)	62 (8.2%)	0.110
Female gender	269 (47.6%)	347 (46%)	0.552
Birth weight (grams)	3260 (2940–3560)	3295 (3020–3595)	0.076
Birth weight percentile	45 (21–74)	50 (27–74)	0.47
Small for gestational age (<10th centile)	67 (11.9%)	87 (11.5%)	0.851
Severe small for gestational age (<3rd centile)	22 (3.9%)	30 (4%)	0.941
Large for gestational age (>90th centile)	68 (12%)	89 (11.8%)	0.891
5-min Apgar score	9.8 (0.8)	9.9 (0.7)	0.268
Neonatal complications	29 (5.1%)	23 (3%)	0.054

Data expressed as *n* (%), median (IQR), or mean (SD).

**Table 5:** Symptoms and diagnosis of SARS-CoV-2 infection and COVID-19 disease in the pandemic cohort based on the level of maternal WHO-5 well-being.

Characteristics	WHO-5 ≤ 52 (*n* = 565)	WHO-5 > 52 (*n* = 755)	*p*-Value
Positive SARS-CoV-2 testing	88 (15.6%)	114 (15.1%)	0.812
Symptoms of SARS-CoV-2 infection within the last 10 weeks	95 (16.8%)	87 (11.5%)	0.006
Fever	25 (4.4%)	19 (2.5%)	0.056
Dry cough	44 (7.8%)	31 (4.1%)	0.004
Difficulty breathing or shortness of breath	17 (3%)	12 (1.6%)	0.082
Diarrhea	20 (3.5%)	16 (2.1%)	0.117
Other respiratory symptoms	9 (1.6%)	8 (1.2%)	0.534
Myalgia	17 (3%)	17 (2.3%)	0.390
Skin rash	5 (0.9%)	4 (0.5%)	0.438
Loss of taste or smell	15 (2.7%)	12 (1.6%)	0.176
Other	10 (1.8%)	16 (2.1%)	0.651
Combination of symptoms predictable for SARS-CoV-2 infection			
At least two symptoms or anosmia	44 (7.8%)	39 (5.2%)	0.052
At least three symptoms or anosmia	22 (3.9%)	20 (2.6%)	0.202
Fever, cough and dyspnea	8 (1.4%)	1 (0.1%)	0.005
Symptom-relatedCOVID-19 severity			
Mild	2 (14.5%)	79 (10.5%)	0.026
Moderate	5 (0.9%)	7 (0.9%)	0.936
Severe	8 (1.4%)	1 (0.1%)	0.005
COVID-19 disease			
Hospital admission for COVID-19 disease	15 (2.7%)	3 (0.4%)	<0.001
Pneumonia	3 (0.5%)	1 (0.1%)	0.192
Severe pneumonia	2 (0.4%)	1 (0.1%)	0.403
Oxygen support	2 (0.4%)	1 (0.1%)	0.403
Admission to intensive care unit	1 (0.2%)	1 (0.1%)	0.837
Invasive ventilatory support	1 (0.2%)	0 (0%)	0.248

Data are expressed as *n* (%). RT-PCR: Real Time Polymerase chain reaction; SARS-CoV-2: severe acute respiratory syndrome coronavirus 2.

**Table 6:** Multivariate analysis of factors associated to poor maternal WHO-5 well-being in the pandemic cohort.

	Univariate Analysis		Multivariate Analysis		
	OR (95% CI)	*p*-value	OR (95% CI)	*p*-Value	Betta Coefficient
Baseline maternal characteristics					
Age (years)	0.98 (0.96–1)	0.051			
Gestational age at recruitment (weeks)	1.04 (1.03–1.05)	<0.001			
Non-European ethnicity	1 (0.8–1.3)	0.977			
Low socio-economic status	1 (0.8–1.3)	0.674			
Tobacco use during pregnancy	0.95 (0.7–1.4)	0.789			
Psychiatric disorders	6.4 (2.4–16.9)	<0.001	7.1 (2.6–19)	<0.001	1.947
Thyroid diseases	0.6 (0.4–1)	0.051			
Nulliparity	1 (0.8–1.3)	0.681			
Assisted reproductive techniques	0.7 (0.5–1.2)	0.208			
Pregnancy outcomes					
Trimester (first vs. third)	1.7 (1.5–1.9)	<0.001	1.7 (1.5–2)	<0.001	0.537
Induction of labor	1.1 (0.9–1.4)	0.353			
Cesarean section	0.99 (0.8–1.3)	0.99			
SARS-CoV-2 status					
Positive SARS-CoV-2 testing	1 (0.8–1.4)	0.812			
Presence of at least one COVID-19 symptom	1.5 (1.1–2.1)	0.006			
Presence of fever, cough and dyspnea	10.8 (1.3–86.8)	0.025			
Presence of severe COVID-19 symptoms	10.8 (1.3–86.8)	0.025			
Hospital admission for COVID-19	6.8 (1.9–23.7)	0.002	4.8 (1.4–16.7)	0.014	1.565
Constant					−1.606

Data are expressed as *n* (%). OR: Odds Ratio; CI: confidence interval; SARS-CoV-2: Severe Acute Respiratory Syndrome Coronavirus 2; COVID-19: Coronavirus 19 disease.

## Data Availability

Data available subject to previous ethics committee agreement.

## Appendix A

### Appendix A.1. COVID-19 Evaluation

COVID-19 symptoms were recorded at hospital admission using a structured self-prepared questionnaire that included questions about risk factors and COVID-19 suggestive symptoms noticed between mid-February 2020 and the time of SARS-CoV-2 testing. All positive women completed the same questionnaire again 4–5 weeks later. Symptomatic SARS-CoV-2 infected women were defined as having at least one of the following symptoms: fever, dry cough, anosmia or ageusia, dyspnea, myalgia, diarrhea, sore throat, skin rash, or discoloration of fingers and/or toes.

### Appendix A.2. Sample Collection

Maternal blood samples were drawn from peripheral veins in first and third trimester participants, at recruitment. Samples were centrifuged at 1500× *g* for 10 min at 4 °C and sera immediately stored at −80 °C until further analysis. For SARS-CoV-2 IgG and IgM/IgA antibody determination, the COVID-19 VIRCLIA^®^ Monotest (Vircell Microbiologist, Granada, Spain) was used. Indeterminate results were re-tested (VITROS^®^ Immunodiagnostic Products Anti-SARS-CoV2 Total Tests, Ortho Clinical Diagnostics, Rochester, NY, USA) and classified as positive or negative. Likewise, results positive for IgM + IgA but negative for IgG in women reporting no symptoms suggestive of COVID-19 during the 10 weeks prior testing were re-tested with Luminex and classified as positive or negative [33]. A serological result was considered positive if any of the following were found: (a) IgG positive, (b) IgM + IgA positive in women with symptomatic COVID-19, (c) IgM + IgA positive confirmed by two tests (Vircell and Luminex).

Nasopharyngeal swab samples for SARS-CoV-2 RNA RT-PCR were collected in all third trimester pregnancies recruited at hospital admittance. Samples were collected in Micronics tubes with Zymo DNA/RNA Shield Lysis buffer. RNA was extracted using the Quick-DNA/RNA Viral MagBead kit (Zymo) and the TECAN Dreamprep robot. Five microliters of RNA solution were added to 15 μL of the rRT-PCR master mix (Luna Universal Probe One-Step RT-qPCR Kit; New England Biolabs) and used for amplification of the SARS-CoV-2 N1 and N2 regions, as well as the human RNase P gene as control, as described in the CDC-006-00019 CDC/DDID/NCIRD/Division of Viral Diseases protocol released 3/30/2020. A SARS-CoV-2 positive result was considered if Ct values for N1, N2, and RNase P were below 40. Samples discordant for N1 and N2 were repeated and samples with a Ct ≥ 40 for RNase P were considered as invalid.

SARS-CoV-2 infection was defined by either a positive serological result or RT-PCR in nasopharyngeal swabs.

## Appendix B

**Table A1:** Baseline characteristics of pre-pandemic and COVID-19 pandemic pregnant women cohorts.

Characteristics	Pre-Pandemic (*n* = 345)	Pandemic (*n* = 1320)	*p*-Value
Ethnicity			
White	279 (80.9%)	858 (65%)	<0.001
Latin American	49 (14.2%)	297 (22.5%)	0.001
Black	6 (1.7%)	23 (1.7%)	0.997
Asian	6 (1.7%)	81 (6.1%)	0.001
Others	5 (1.4%)	61 (4.6%)	0.007
Tobacco use during pregnancy	27 (7.8%)	127 (9.6%)	0.305
Pre-pregnancy BMI (kg/h^2^)	23.8 (4.8)	24.1 (4.7)	0.29
Medical history			
Obesity (BMI > 30)	39 (11.3%)	157 (11.9%)	0.762
Psychiatric disorders *	15 (4.3%)	28 (2.1%)	0.020
Thyroid diseases	31 (9%)	91 (6.9%)	0.184
Obstetric history			
Nulliparous	203 (58.8%)	725 (54.9%)	0.192

Data are expressed as *n* (%) or median (IQR) or mean (SD). BMI: Body mass index. * Psychiatric disorders requiring therapy during pregnancy.

**Table A2:** Self-administered questionnaire on COVID-19 pandemic-related conditions.

Characteristics	Total Cohort (*n* = 480)	WHO-5 ≤ 52	WHO-5 >52	*p*-Value
SARS-CoV-2 diagnosis by laboratory test				0.079
Yes	7 (1.5%)	10 (3.4%)	2 (1%)	
No	473 (98.5%)	287 (96.6%)	207 (99%)	
Contact with a symptomatic SARS-CoV-2 person				0.098
Yes	42 (8.8%)	21 (7%)	24 (11.2%)	
No	438 (91.3%)	278 (93%)	190 (88.8%)	
Know someone diagnosed by SARS-CoV-2				0.247
Yes	129 (26.9%)	74 (24.4%)	62 (29%)	
No	351 (73.1%)	229 (75.6%)	152 (71%)	
Degree of concern about SARS-CoV-2 epidemic				0.088
I’m very worried	192 (40%)	112 (37.2%)	94 (44.1%)	
I’m quite worried	222 (46.3%)	141 (46.8%)	97 (45.5%)	
I’m a little worried	59 (12.3%)	45 (15%)	18 (8.5%)	
Don’t care	7 (1.5%)	3 (1%)	4 (1.9%)	
Worry of getting the disease yourself or a family member				0.537
I’m very worried	279 (58.1%)	170 (56.1%)	133 (62.1%)	
I’m quite worried	159 (33.1%)	107 (35.3%)	63 (29.4%)	
I’m a little worried	40 (8.3%)	25 (8.3%)	17 (7.9%)	
Don’t care	2 (0.4%)	1 (0.3%)	1 (0.5%)	
Effect on the pregnancy and fetus concerns				0.220
I’m very worried	332 (69.2%)	202 (66.9%)	156 (72.9%)	
I’m quite worried	84 (17.5%)	58 (19.2%)	32 (15%)	
I’m a little worried	53 (11%)	33 (10.9%)	24 (11.2%)	
Don’t care	11 (2.3%)	9 (3%)	2 (0.9%)	
Personal economic concern				0.944
I’m very worried	226 (47.1%)	146 (48.2%)	102 (47.7%)	
I’m quite worried	148 (30.8%)	88 (29%)	66 (30.8%)	
I’m a little worried	86 (17.9%)	55 (18.2%)	38 (17.8%)	
Don’t care	20 (4.2%)	14 (4.6%)	8 (3.7%)	
Impact on global economy concerns				0.110
I’m very worried	199 (41.5%)	124 (40.9%)	93 (43.5%)	
I’m quite worried	198 (41.3%)	116 (38.3%)	94 (43.9%)	
I’m a little worried	72 (15%)	55 (18.2%)	24 (11.2%)	
Don’t care	11 (2.3%)	8 (2.6%)	3 (1.4%)	
Excessive worrying				0.092
Yes	41 (8.5%)	36 (11.9%)	14 (6.5%)	
No	439 (91.5%)	267 (88.1%)	200 (93.5%)	
Does the pregnant woman have enough information regarding the effects of the virus on pregnancy and the fetus				0.332
Yes	216 (45%)	141 (47-2%)	89 (41.6%)	
No	264 (55%)	160 (52.8%)	125 (58.4%)	
Isolation in primary residence				0.515
Yes	448 (93.3%)	277 (91.4%)	199 (93%)	
No	32 (6.7%)	26 (8.6%)	25 (7%)	
People at risk living at home				0.548
Yes	46 (9.6%)	27 (8.9%)	23 (10.8%)	
No	434 (90.4%)	275 (91%)	189 (89.2%)	
Terrace or garden at home				0.809
Yes	251 (52.3%)	158 (53.2%)	111 (52.1%)	
No	229 (47.7%)	139 (46.8%)	102 (47.9%)	
Work				0.748
No	419 (87.3%)	266 (88.1%)	184 (86%)	
Yes, from home	51 (10.6%)	29 (9.6%)	25 (11.7%)	
Yes, at my usual place of work	10 (2.1%)	7 (2.3%)	5 (2.3%)	
How many times a week does she go out				0.352
Never	162 (33.8%)	97 (32%)	78 (36.4%)	
One or two times a week	232 (48.3%)	146 (48.2%)	106 (49.5%)	
Between three and five times a week	52 (10.8%)	36 (11.9%)	19 (8.9%)	
Six or more times a week	34 (7.1%)	24 (7.9%)	11 (5.1%)	
Coping with isolation				<0.001
Very well	94 (19.6%)	69 (23.1%)	28 (13.1%)	
Pretty well	309 (64.4%)	197 (65.9%)	136 (63.8%)	
Poorly	68 (14.2%)	29 (9.7%)	42 (19.7%)	
Very poorly	9 (1.9%)	4 (1.3%)	7 (3.3%)	
Mental health before the pandemic				0.069
Excellent	106 (26.6%)	75 (29.8%)	38 (21.5%)	
Very good	180 (45.2%)	115 (45.6%)	81 (45.8%)	
Good	97 (24.4%)	56 (22.2%)	46 (26%)	
Regular	11 (2.8%)	4 (1.6%)	10 (5.6%)	
Bad	4 (1%)	2 (0.8%)	2 (1.1%)	

Data are expressed as *n* (%). SARS-CoV-2: severe acute respiratory syndrome coronavirus.

**Table A3:** Prevalence of SARS-CoV-2 infection during pregnancy.

Characteristics	Total Cohort (*n* = 1320)
SARS-CoV-2 positive (RT-PCR and/or Ab)	202 (15.3%)
First trimester	82 (40.6%)
Third trimester	120 (59.4%)
RT-PCRa positive	26 (3%)
Ab for SARS-CoV-2 infection IgM/A/G	
Negative	1120 (84.8%)
Positive	200 (15.2%)

Data are expressed as *n* (%) or median (IQR); SARS-CoV-2: severe acute respiratory syndrome coronavirus; RT-PCR: Real Time Polymerase chain reaction; Ab: Antibody. Data available only for 876 cases (Third trimester participants).
